# The Enzymatic and Metabolic Capabilities of Early Life

**DOI:** 10.1371/journal.pone.0039912

**Published:** 2012-09-10

**Authors:** Aaron David Goldman, John A. Baross, Ram Samudrala

**Affiliations:** 1 Department of Ecology and Evolutionary Biology, Princeton, New Jersey, United States of America; 2 School of Oceanography, University of Washington, Seattle, Washington, United States of America; 3 Center for Astrobiology and Early Evolution, University of Washington, Seattle, Washington, United States of America; 4 Department of Microbiology, University of Washington, Seattle, Washington, United States of America; Institute of Infectious Disease and Molecular Medicine, South Africa

## Abstract

We introduce the concept of metaconsensus and employ it to make high confidence predictions of early enzyme functions and the metabolic properties that they may have produced. Several independent studies have used comparative bioinformatics methods to identify taxonomically broad features of genomic sequence data, protein structure data, and metabolic pathway data in order to predict physiological features that were present in early, ancestral life forms. But all such methods carry with them some level of technical bias. Here, we cross-reference the results of these previous studies to determine enzyme functions predicted to be ancient by multiple methods. We survey modern metabolic pathways to identify those that maintain the highest frequency of metaconsensus enzymes. Using the full set of modern reactions catalyzed by these metaconsensus enzyme functions, we reconstruct a representative metabolic network that may reflect the core metabolism of early life forms. Our results show that ten enzyme functions, four hydrolases, three transferases, one oxidoreductase, one lyase, and one ligase, are determined by metaconsensus to be present at least as late as the last universal common ancestor. Subnetworks within central metabolic processes related to sugar and starch metabolism, amino acid biosynthesis, phospholipid metabolism, and CoA biosynthesis, have high frequencies of these enzyme functions. We demonstrate that a large metabolic network can be generated from this small number of enzyme functions.

## Introduction

The expansion of genomic and biomolecular data has led to large repositories of genetic sequences [1], [2], protein structures [3], [4], protein functions [1], [5], [6], networks of metabolic reactions [6], [7], and networks of molecular interactions [7]. By comparing these features across the universal tree of known life, it is possible to reveal traits that are common to a broad range of organisms and, therefore, are likely to have been present in the Last Universal Common Ancestor (LUCA) and its predecessors [8]. In this study, we focus on three such approaches with distinctly different methodologies.

Harris *et al.* [9] performed a universal sequence comparison by identifying Clusters of Orthologous Groups of genes (COGs) [10] that were present in all completely sequenced genomes. Of the 3100 groups catalogued in the COG database, 80 are common across all of the genomes available at the time. Because any evolutionary pressure must act primarily on sequence, the appearance of these COGs across all sequenced genomes can be directly attributed to the presence of related genes in the genome of LUCA. Early horizontal gene transfers may make a COG look more ancient by this method, while gene losses, even recent ones, will make a COG look less ancient.

The work of the Caetano-Anollés group [11]–[13] has provided a second type of comparative bioinformatics analysis pertaining to protein structure. Protein structure may be the most highly conserved feature of molecular evolution [14], [15] because the development of a new protein structure is unlikely [16], [17], whereas the repurposing of an existing protein structure is simple by comparison [18], [19]. Their method begins with a survey of the distribution of protein fold architectures across all available sequenced genomes. These distributions are then used to create a phylogeny of all protein fold architectures. This method assumes that both broad taxonomic distribution and genomic recurrence are quantitatively related to ancestry. Wang *et al.*, [16] also identified a specific branch node that represents the divergence of LUCA into three domains of life. In their phylogeny, 165 protein fold architectures are deeper branching than the divergence of LUCA and are, thus, considered to have been present in the proteome of LUCA. Because fold architecture is more highly conserved than gene sequence, this phylogeny may represent the deepest view of ancient evolution to date. On the other hand, it is not certain that every fold architecture is the result of a single evolutionary origin [20].

Another substantially different comparative bioinformatics analysis was performed by Srinivasan and Morowitz [21] in which metabolic reactions were surveyed across organisms without any reliance on enzyme sequence or structure data. To do so, the authors superimposed entries stored in the Kyoto Encyclopedia of Genes and Genomes reactions database (KEGG) [6] for the five autotrophic organisms, four bacteria and one archaean, with extensive available metabolic network data. Two hundred eighty-six reactions were identified as common among all five organisms and were thus assumed to have been present in LUCA. It should be noted that the authors assumed an autotrophic origin of life and that this analysis was limited accordingly to data from only autotrophic organisms. The function of a particular protein family is perhaps the least conserved feature of molecular evolution [15], [19], [22]. However, a protein may evolve a new enzymatic function and replace an unrelated protein with the same function through the process of non-orthologous gene displacement [22]. Thus the conservation of the presence of an enzymatic function may be high, even if the protein family that imparts that function changes during the course of evolution.

While the gene content of modern organisms provides strong evidence for a common ancestor [8], [23] it is not clear that the root of the tree of life represents a single organism or a community of organisms exhibiting rampant horizontal gene transfer [24], [25]. Furthermore, it is not clear what effects early horizontal gene transfer, non-orthologous gene displacement, or other mechanisms of gene gain and loss have on comparative bioinformatics analyses such as those outlined above. Gene sequence, protein structure, and enzyme function represent separate, but related, features of cellular biology and, consequently, respond differently to evolutionary selection pressures [15]. Each of these methods should not only reveal different features of LUCA, but should also do so with a unique degree of confidence and sensitivity. Here we compare the results of these three studies in order to make high confidence predictions through “metaconsensus” (*i.e.* a consensus between these independent consensus methods). The results are used to determine a higher confidence minimal catalytic repertoire of LUCA and to extrapolate the metabolic properties produced by these catalytic functions.

## Results and Discussion

### Metaconsensus enzymes and their functions

In order to compare the three aforementioned datasets to one another, the contents of each were converted to 3-letter Enzyme Commission (EC) codes (See methods; Data S1). We henceforth refer to the converted datasets as the universal sequence (Harris *et al.* [9]), universal structure (Kim *et al.* [26]) and universal functions (Srinivasan and Morowitz [21]). The universal sequence dataset contains 12 EC groups, the universal structure dataset contains 155 EC groups, and the universal reaction dataset contains 53 EC groups.

**Figure 1:**
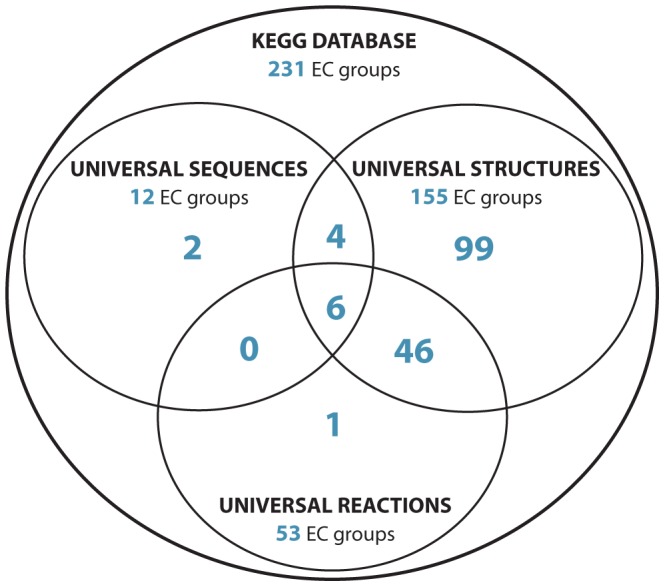
Metaconsensus analysis of conserved enzyme functions identified by three independent comparative bioinformatics methods. These methods include comparisons of clusters of orthologous protein sequences [9], protein fold architectures [26], and metabolic reactions [21]. Members in each of these datasets are converted to enzyme functions represented by a three-term EC code and the resulting datasets are made nonredundant. Six metaconsensus enzyme functions are found in all three datasets and thus are very likely to have been present in LUCA. Because the universal reactions data were acquired by comparing only autotrophic organisms, this analysis may be overly dependent on whether or not LUCA was also autotrophic. Thus, the four EC groups common between the universal sequence and universal structure datasets, but not present in the universal reaction dataset, are also likely to have been present in LUCA.

Six enzyme functions are found in all three datasets (Figure 1). Four additional enzyme functions are found in the universal sequence and universal structure datasets, but not the universal reaction dataset. Note that the universal reaction dataset was derived solely from autotrophic organisms and thus its relevance to LUCA may be dependent on LUCA having been autotrophic. We consider these four additional enzyme functions along with the six metaconsensus enzyme functions in the following analyses. Three of the six EC groups found in all three datasets are transferases. The other three are an oxidoreductase, a lyase, and a ligase. The four EC groups found in both the universal sequence and universal structure datasets, but not in the universal reaction dataset, are all hydrolases. Srinivasan and Morowitz's emphasis on autotrophy may have excluded these hydrolase functions, which are catabolic by definition. Details of these enzyme functions are presented in Table 1.

**Table 1:** Metaconsensus enzyme functions and their associated metal and nucleotide cofactors.

*Consensus*	*EC group*	*Enzyme description (abridged from EC)*	*Metal cofactors*	*Nucleotide-derived cofactors*
Universal Sequence, Structure, and Function	1.3.1.-	Oxidoreductases. Acting on the CH-CH. NAD(+) as acceptor	Fe-S	FAD, FMN
	2.4.1.-	Transferases. Glycosyltransferases. Hexosyltransferases.	Ca, Mg, Mn	None
	2.7.1.-	Transferases. Transfering P groups. Alcohol acceptor.	Ca, Mg	None
	2.7.7.-	Transferases. Transfering P groups. Nucleotidyltranferases	Co, Mg	None
	4.1.2.-	Lyases. C-C bonds. Aldehyde-lyase.	Zn, Mg, Mn	FAD
	6.3.2.-	Ligases. C-N bonds. D-amino acid ligases.	Mg, Mn	None
Universal Sequence and Structure	3.1.2.-	Hydrolases. Ester bonds. Thiolester hydrolases.	Zn, Mg	None
	3.1.4.-	Hydrolase. Ester bonds. Phosphoric diester hydrolases.	Heme, Zn, Mg	None
	3.2.1.-	Hydrolase. Glycosylases. Glycosidases	Ca	None
	3.5.1.-	Hydrolases. C-N bonds (nonpeptide). Linear amides.	Ni, Co	ATP

We corroborate the antiquity of these metaconsensus enzyme functions by the association with both metal and nucleotide derived cofactors [27]. It is thought that many catalytic mechanisms of metalloenzymes may have originated during the transition from prebiotic chemistry to genetically directed metabolism [28]–[30]. Early metalloproteins likely tended to use structures that were ambiguous rather than specific in the metals that they bound [30], [31]. Similarly, nucleotide derived cofactors are believed to reflect a preceding state in which the same reaction was catalyzed by a ribozyme [27], [32].

According to Uniprot annotations [1], all of the ten conserved EC groups have metal cofactors coupled to the catalytic mechanisms of enzymes within the group. These results may reflect previous work implicating iron-sulfur clusters [33], [34] and zinc [35], [36] as having important catalytic functions in prebiotic synthesis reactions, as well as an important early role for divalent cations such as Mg^2+^. Only three of the ten conserved EC groups also use nucleotide-derived cofactors, although it should be noted that uses of nucleotides and nucleotide-derived coenzymes like CoA as substrates was excluded from this analysis.

Ancestry values [26] were used to identify the most ancient folds defined by SCOP [4] associated with each metaconsensus enzyme function. The ancestry value, developed by the Caetano-Anolles group [37], is a scale of relative evolutionary age where 0% is the most ancient fold architecture and 100% is the most recent fold architecture. The establishment of the DNA genome is constrained at 19% ancestry by the appearance of folds that catalyze the critical ribonucleotide reductase step in deoxyribonucleotide synthesis [38], [39]. The evolutionary divergence of LUCA can be constrained at 40% ancestry by the first appearance of a fold found exclusively in a single domain of life [16].

**Figure 2:**
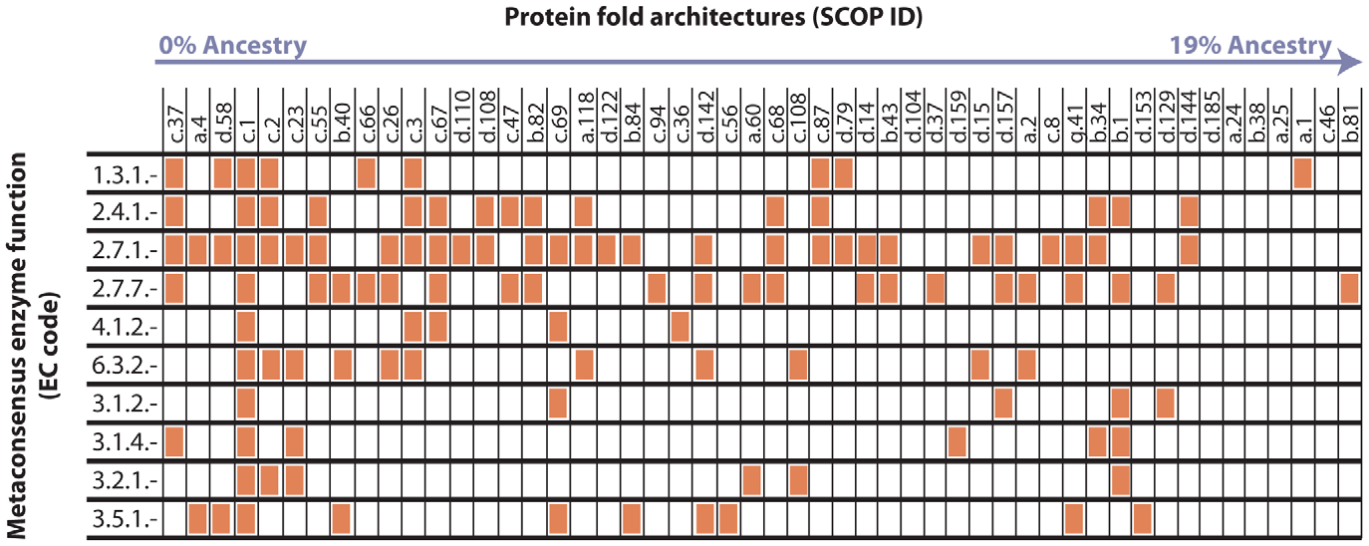
Ancestral folds associated with metaconsensus enzyme functions. Folds are given in the horizontal axis by their SCOP code [4]. Orange boxes indicate associations with metaconsensus enzyme reactions in the vertical axis. These folds (with ancestry values between 0% and 19%) were previously predicted to have originated before the establishment of the DNA genome [38]. All of the metaconsensus enzyme functions are associated with a number of ancient folds. The three transferases (EC codes 2.4.1.-, 2.7.1.-, and 2.7.7.-) are associated with the highest number of these ancient folds.

Figure 2 shows that many ancient folds are associated with these ten metaconsensus enzyme functions. All ten metaconsensus enzyme functions can be catalyzed by the triosephosphate isomerase (TIM) beta/alpha barrel (SCOP ID = c.1; ancestry = 1.9%). The TIM beta/alpha barrel is a very versatile fold architecture that is able to catalyze a number of disparate enzyme functions [40]. Other ancestral catalytic folds common among these EC groups include the Ferredoxin-like fold (SCOP ID = d.58, ancestry = 1.3%), the Ribonuclease H-like motif (SCOP ID = c.55; ancestry = 3.7%), the S-adenosyl-L-methionine-dependent methyltransferase fold (SCOP ID = c.66; ancestry = 5.0%), the Adenine nucleotide alpha hydrolase-like fold (SCOP ID = c.26; ancestry = 5.7%), the UDP-glycosyltransferase/glycogen phosphorylase fold (SCOP ID = c.87; ancestry = 11.9%), and the Globin-like fold (SCOP ID = a.1; ancestry = 18.8%).

Most proteins are composed of more than one fold within a single peptide chain. In many cases, some ancient folds associated with an EC group are not catalytic domains, themselves. By ancestry value, the P-loop containing nucleoside triphosphate fold (SCOP ID = c.37; ancestry = 0.0%) is the most ancient fold associated with any metaconsensus enzyme function, but this fold catalyzes NTP hydrolysis or NDP phosphorylation that is coupled to the enzyme rather than the specific catalytic function of the enzyme. Other ancient folds associated with metaconsensus enzyme functions that do not confer specific catalysis include the DNA/RNA-binding 3-helical bundle (SCOP ID = a.4, ancestry 0.6%), the NAD(P)-binding Rossmann fold (SCOP ID = c.2, ancestry = 2.5%), and the Oligonucleotide/oligosaccharide binding (OB) fold (SCOP ID = b.40; ancestry = 4.4%), to name a few. In combination with the ancestral catalytic folds, these folds probably allowed ancient enzymes to bind a range of substrates and cofactors and couple reactions to NTP hydrolysis. The combinatorial power of these ancient folds could conceivably produce a robust metabolism in LUCA. The prevalence of nucleotide and nucleic acid binding folds associated with metaconsensus enzymes suggests a strong connection to a preceding RNA world scenario.

### Metabolic implications of metaconsensus enzyme functions

**Figure 3:**
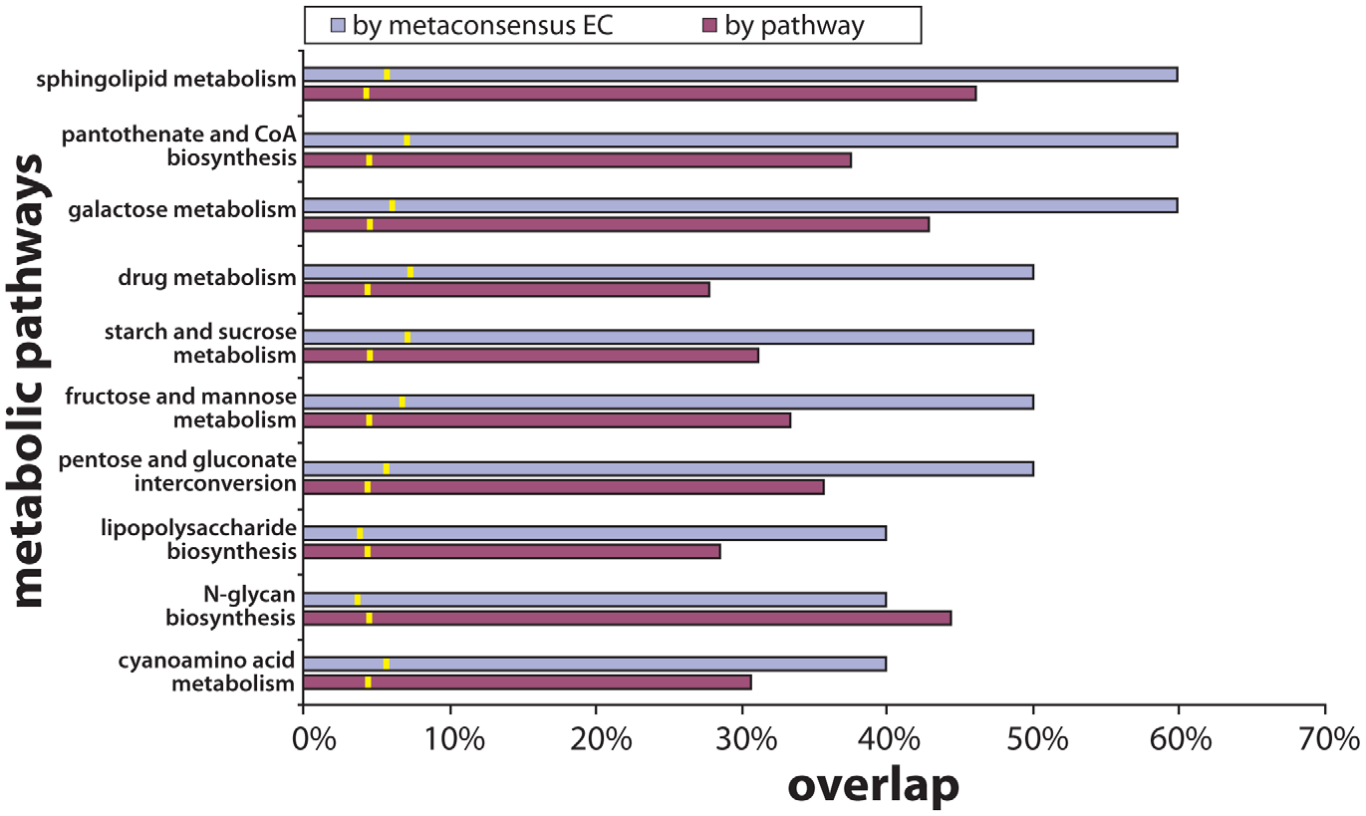
Modern metabolic pathways with the highest frequencies of metaconsensus enzyme functions. Pathways were identified as ancient through a survey of all pathways stored in the KEGG database [6]. For each pathway, the frequency of metaconsensus enzyme functions is presented as both a percentage of the list of pathway enzymes and a percentage of the list of metaconsensus enzyme functions. A negative control is represented by yellow bars, which indicate the average value of pathway enzymes randomly assigned from all enzyme functions in the KEGG database. This analysis identifies amino acid, phospholipid, CoA, and carbohydrate metabolisms as ancient. Diagrams of these pathways with highlighted metaconsensus enzyme functions are available as Figure S1.

Having identified these metaconsensus EC groups, we were able to evaluate the antiquity of modern metabolic pathways based on the presence of these conserved enzyme functions. We performed a survey of enzymes across all metabolic pathways defined by KEGG [6] (Data S2 and Data S3). The percentages of metaconsensus EC groups within each pathway is presented in Figure 3 and the metaconsensus enzyme functions are highlighted on KEGG pathway images in Figure S1. This analysis reveals a list of conserved metabolic pathways that carry out the synthesis and degradation of important biomolecules ranging in size from CoA and simple sugars to N-glycans and sphingolipids.

The high frequency of conserved EC groups in sphingolipid metabolism is interesting because sphingolipids are found in eukaryotes and some bacteria, but are not taxonomically universal [41], [42]. This result may reflect a conserved core of phospholipid metabolism that sphingolipid metabolism happens to resemble. Within the sphingolipid metabolism pathway, metaconsensus enzymes are most closely associated with the conversion of ceramides to sphingosines (Figure S1). The high frequency of conserved EC groups that carry out pantothenate and CoA biosynthesis reflects the universality of CoA in the central metabolisms of organisms. It has been proposed that acetyl CoA was a key constituent of prebiotic synthesis [43]–[46]. An alternate, but not mutually exclusive, explanation is that CoA, as a nucleotide-derived cofactor, is a remnant of ribozyme catalyzed reactions that preceded modern metabolism [32]. The appearance of drug metabolism is curious, although most metaconsensus enzyme functions in this category are involved in fluorouracil metabolism, and may reflect a propensity toward nucleobase chemistry in general (Figure S1). It is important to note that all of these pathways are modern and that their use of metaconsensus enzymes probably does not identify their current configurations as ancient, but rather, illustrates the general metabolic priorities of LUCA.

**Figure 4:**
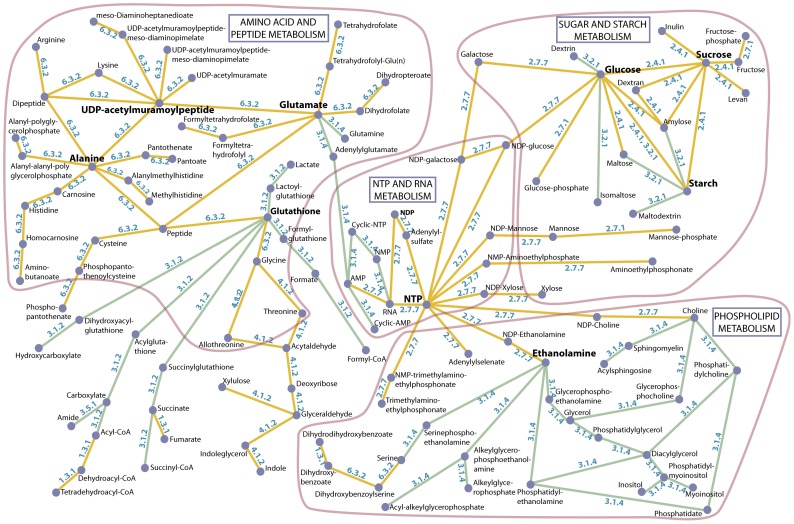
A reconstructed metabolism composed of reactions imparted by metaconsensus enzyme functions. Nodes represent reactants and products while the edges connecting them represent metaconsensus enzyme functions. The network is composed of 119 nodes and 135 edges. Reactions were assembled from the KEGG reactions database and small molecules and cofactors were removed. Yellow edges represent metaconsensus enzyme functions predicted by the universal sequence, universal structure, and universal reaction datasets. Green edges represent metaconsensus enzyme functions predicted by the universal sequence and universal structure datasets, but not the universal reaction dataset. Subnetworks circled in red roughly reflect subsets of metabolism related to amino acids and peptides, nucleotides and RNA, sugars and starches, and phospholipids. This reconstructed metabolism demonstrates that significant metabolic complexity is possible with only these ten metaconsensus enzyme functions.

To extend our analysis beyond the constraints of modern pathways, we reconstructed a representative ancient metabolism using only the metaconsensus enzyme functions. These ten enzyme functions perform nearly three hundred reactions as defined in the KEGG reactions database [6] (Data S4). After generating networks based on these reactions, we identified a relatively large network composed of 119 nodes and 135 edges (Figure 4). The major hub nodes (defined here as nodes connected to five or more edges) include nucleotide triphosphate, glucose, sucrose, starch, glutamate, alanine, glutathione, and UDP-acetylmuramoyl peptide. These hub nodes are circumscribed by subnetworks related to NTP phosphorylation/dephosphorylation and RNA synthesis/degradation, sugar synthesis and starch polymerization/degradation, amino acid synthesis/interconversion, phospholipid synthesis/degradation, and enzymatic peptide synthesis/degradation.

This reconstructed ancient metabolism includes component subnetworks spanning the breadth of modern central metabolism, from monomer synthesis and interconversion, to the synthesis of RNA, proteins, starches, and phospholipids. Even though it is thought that LUCA had a DNA genome [8], this network does not include DNA synthesis. DNA polymerase is included in the general metaconsensus enzyme function (EC 2.7.7.-), but the reduction of ribonucleotides to form deoxyribonucleotides (EC 1.17.4.1) is not a metaconsensus function. The ribonucleotide reductase enzyme is thought to have been the limiting enzyme function in establishing a DNA genome [39] during the development of LUCA from the RNA-protein world. DNA replication does not exhibit a conserved universal core of enzymes, although excision repair DNA polymerases [47], [48] and some RNA polymerase catalytic domains [8], [49] are universally distributed across the tree of life.

It is also curious that the reconstructed metabolism includes enzymatic peptide synthesis. We have previously shown that the translation system reached a modern level of sophistication during the RNA-protein stage of early evolution [38]. Perhaps enzymatic peptide synthesis supplemented this system as new amino acids became available, but before these amino acids were incorporated into the translation system. Alternatively, these enzyme functions may have been involved in the production of peptide-derived biomolecules as they often are now. Taken together, the presence of ancient nucleic acid/nucleotide binding folds in metaconsensus enzymes, the absence of DNA synthesis from the reconstructed metabolic network, and the presence of enzyme functions related to peptide synthesis in the reconstructed metabolic network, indicate that our metaconsensus method is revealing trends more ancient than the divergence of LUCA, perhaps perhaps from the time of the development of the RNA-protein system. Previous bioinformatics-based studies have also found a propensity of ancient enzymes that corroborate an RNA-protein stage of early life [8], [50].

A substantial and general conclusion from this work is that a large metabolic network can be produced with only these ten metaconsensus enzyme functions. This observation demonstrates the strength of the patchwork model of primitive metabolism in which a small number of functionally ambiguous enzymes can generate the groundwork for a complex metabolism [51], [52]. Furthermore, the enzyme functions, themselves, can be produced by variants of one protein fold that imparts the direct enzymatic function and several auxiliary catalytic and noncatalytic protein folds, all of which appeared early in the development of genetically encoded proteins. Thus, whatever the catalytic repertoire of early life, we assert that a relatively complex metabolism can be produced from a small number of enzymatic functions.

## Methods

### Identifying and analyzing conserved enzymes

The dataset of universal COGs was copied directly from Table 1 of Harris *et al.* [9]. The dataset of universal protein structures was downloaded from the MANET database (http://www.manet.uiuc.edu/) [26]. The dataset of universal metabolic reactions was downloaded from the supplemental online information of Srinivasan and Morowitz [21]. The universal structure dataset uses folds from the MANET database with ancestry values ≤0.399, which are considered to have been present in LUCA [16]. EC codes for COGs were identified by searching annotations on the COG database (http://www.ncbi.nlm.nih.gov/COG/) [10]. EC codes for protein fold architectures were extracted from the MANET data file. EC codes for metabolic reactions were identified by searching the KEGG database (http://www.genome.jp/kegg/) [6]. A protein was not included in this analysis if no EC code was available, either because the protein is non-enzymatic or it is an enzyme that has not yet been incorporated into the EC system. Thus, our analysis is limited to enzyme-mediated metabolism and is less likely to incorporate recently discovered enzyme functions.

In the resulting datasets, the final (fourth) term of each EC code was deleted in order to remove a layer of specificity from the conserved enzyme functions. This final term is defined by the Nomenclature Committee of the International Union of Biochemistry and Molecular Biology (NC-IUBMB) as the “serial number of the enzyme in its sub-subclass“. For example the sub-subclass of EC 1.2.3.- is [Oxidoreductase. Acting on the aldehyde or oxo group of donors. With oxygen as acceptor]. The serial number 1.2.3.4 designates oxalate oxidase, while the serial number 1.2.3.5 designates glyoxylate oxidase, a very a similar reaction. Our generalization of enzyme functions is consistent with the patchwork hypothesis of early metabolic pathway evolution [51], [52].

These sets of conserved enzyme functions were made nonredundant by removing all but one instance of repeated 3-term EC codes. The ancestry of protein fold architectures associated with these ten conserved enzymes were extracted from the MANET data file [53]. These folds were identified as catalytic or associated noncatalytic folds by their SCOP database functional annotations [4]. Metal and nucleotide derived cofactors associated with each conserved enzyme function were identified by surveying Uniprot annotations [1] for proteins with metaconsensus EC classifications.

### Analysis of metabolic pathways

Lists of metabolic pathways and their associated enzymes were downloaded from the KEGG FTP site (ftp://ftp.genome.jp/pub/kegg/). These pathway lists were generalized to 3-term EC codes and made nonredundant in the same manner described previously for metaconsensus enzyme datasets. Percentages of conserved enzymes were calculated with respect to the total number of conserved enzymes and the total number of enzyme groups within each pathway to produce Figure 3. The same list of metabolic reactions was used to reconstruct a network of reactions imparted by only the metaconsensus enzyme functions. Small molecules such as H_2_O and CO_2_ were removed from the reactions as these would impose false connectivity between reactants and products. Similarly, the conversion of ATP to ADP was removed from reactions when its presence was only attributable to energetic coupling and did not otherwise contribute to the reaction product. Networks were generated with reactants and products as nodes and the enzyme functions that catalyze them as edges. The resulting networks were visualized using Osprey [54]. The 119-node network presented in Figure 4 was identified visually. Nearly all other networks were 3 to 5 nodes in size. The 119-node network was hand annotated and relabeled for clarity using graphics editors.

## Supporting Information

Figure S1 Diagrams of pathways from Figure 3 with metaconsensus enzyme functions highlighted in red. Pathway diagrams were adapted from KEGG pathway maps [6] with permission from the KEGG database managers.
(PDF)

Data S1 A text file containing nonredundant lists of generalized enzyme functions (by Enzyme Commission code) that are predicted to be ancient by previous comparative bioinformatics studies.
(TXT)

Data S2 A text file containing nonredundant lists of generalized enzyme functions (by Enzyme Commission code) in metabolic pathways defined by the KEGG database.
(TXT)

Data S3 A text file listing percentage overlap between the metaconsensus enzyme set and metabolic pathways defined by the KEGG database.
(TXT)

Data S4 A text file listing all reactions stored in the KEGG database that are catalyzed by metaconsensus enzyme functions.
(TXT)
