# Supplementary figures and images for: The Enzymatic and Metabolic Capabilities of Early Life
Source: PLoS One. 2012 Sep 10;7(9):e39912. doi: 10.1371/journal.pone.0039912 (PMC3438178; doi:10.1371/journal.pone.0039912)

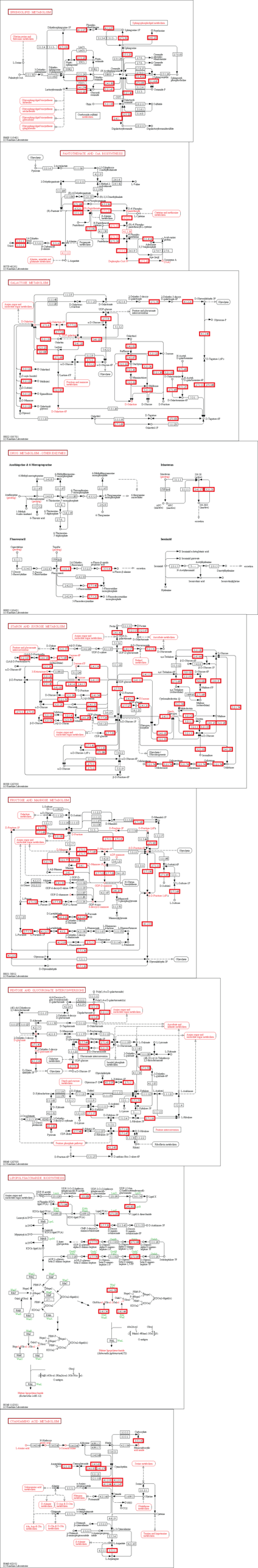

Supplement: Figure S1 — Diagrams of pathways from Figure 3 with metaconsensus enzyme functions highlighted in red. Pathway diagrams were adapted from KEGG pathway maps [6] with permission from the KEGG database managers. (PDF) [file pone.0039912.s001.pdf]
